# The reliability and validity of the short version of the WHO Quality of Life Instrument in an Arab general population

**DOI:** 10.4103/0256-4947.51790

**Published:** 2009

**Authors:** Jude U. Ohaeri, Abdel W. Awadalla

**Affiliations:** a Department of Psychiatry, Psychological Medicine Hospital, Safat, Kuwait; b Department of Psychiatry, Faculty of Medicine, Kuwait University, Jabriya, Kuwait

## Abstract

**BACKGROUND AND OBJECTIVES:**

There is rising interest in quality of life (QOL) research in Arabian countries. The aim of this study was to assess in a nationwide sample of Kuwaiti subjects the reliability and validity of the World Health Organization Quality of Life (WHOQOL-BREF), a shorter version of the widely used QOL assessment instrument that comprises 26 items in the domains of physical health, psychological health, social relationships, and the environment.

**METHODS:**

A one-in-three systematic random proportionate sample of consenting Kuwaiti nationals attending large cooperative stores and municipal government offices in the six governorates completed the Arabic translation of the questionnaire. The indices assessed included test-retest reliability, internal consistency, item internal consistency (IIC), item discriminant validity (IDV), known-groups and construct validity.

**RESULTS:**

There were 3303 participants (44.8% males, 55.2% females, mean age 35.4 years, range 16 to 87 years). The intra-class correlation for the test-retest statistic and the internal consistency values for the full questionnaire and the domains had a Cronbach's alpha≥0.7. Of the 24 items that constitute the domains, 21 met the IIC requirement of correlation ≥0.4 with the corresponding domain, while 16 met the IDV criterion of having a higher correlation with their corresponding domain than other domains. Domain scores discriminated significantly between well and sick groups. In the factor analysis, four strong factors emerged with the same construct as in the WHO report.

**CONCLUSION:**

The Arabic translation of the WHOQOL-BREF has impressive reliability and validity indices. The poor IDV findings are due to the multidimensional nature of the questionnaire. The highly significant validity indices should reassure researchers that the questionnaire represents the same constructs across cultures. Negatively worded items possibly need refinement.

Recent interest in quality of life (QOL) as an outcome measure in medicine has inspired studies in chronic medical^1^–^3^ and general population subjects^4^,^5^ in Arab and neighboring countries. To further this interest, it is important to use assessment instruments that are psychometrically sound and have cross-cultural validity, so as to make findings comparable across countries. In this regard, the short version of the World Health Organization's QOL Instrument, the WHOQOL-BREF,^6^ is of interest for the following reasons: First, it was simultaneously developed in diverse cultures, thus overcoming the usual controversy over the problem of applying a questionnaire articulated in one culture in a different culture.^7^ This means that the instrument has a strong potential for easy cross-cultural applicability, since the items are framed in culture-neutral terminology. Second, the items include widely valued contextual factors of life that are not generally regarded as health-related.^8^ Therefore, it is a generic instrument that assesses health-related QOL (HRQOL), and social, environmental and subjective well-being issues.

The Arabic translation of the WHOQOL-BREF has been shown to have highly significant structural integrity characteristics.^9^ The Persian translation has been shown to have significant values for some reliability and validity indices.^4^ In this study, we sought to contribute to published data on the Arab population by sampling Kuwaiti subjects. Our specific objectives were to assess the following reliability and validity indices, in comparison with the WHO 23-country report:^6^

(i) the test-retest reliability of the WHOQOL-BREF; (ii) the floor/ceiling effect and acceptability of the WHOQOL-BREF, as well as the internal consistency of the full questionnaire and its constituent four domains of QOL; (iii) the item-internal consistency (IIC) and item-discriminant validity (IDV);^10^ (iv) the construct validity by exploratory factor analysis, and known-groups validity by analyzing the differences in domain scores between sick and well subjects. Based on published information,^4^,^6^,^9^ we hypothesized that the reliability and validity indices in an Arab population would be consistent with these indices in other populations. Furthermore, all domains were expected to be strongly and positively correlated with the concept of overall QOL and health (i.e., general facet on health and QOL).^6^

## METHODS

Of the 3.4 million population of Kuwait, Kuwaiti nationals make up 1.1 million (48.9% male, 51.1% female) (2007 census). About 97% live in urban areas. The adult literacy rate in Arabic is 83.5% (2003 estimate) and the unemployment rate is 2.3% (2004 estimate by the Kuwait Public Authority for Civil Information). Administratively, the country is divided into six governorates, each consisting of a centrally located large cooperative supermarket store, as well as municipal government and immigration offices. Our sampling framework was the six governorates, and participants were recruited at the above locations. Our sample size was guided by the recommendation of the International Quality of Life Assessment (IQOLA) project researchers, that the sample size for general population norming should be 2500-3000.^10^

We obtained census data for each governorate from the Public Authority for Civil Information. Using quota sampling, we generated the number of potential participants that would constitute a proportionate representation of governorates, by gender, for a sample size of at least 3000 subjects. Although Kuwait has the infrastructure for generating a sampling framework for a representative general population study (e.g. telephone listing, voting register), the strict conservative culture makes it impossible to carry out house-to-house surveys for private studies such as ours. To overcome this obstacle, researchers have attempted to target people that visit popular government administrative offices (e.g. municipal government and immigration offices) and the large cooperative shops. In a recent Kuwaiti study,^11^ it was found that the large cooperative shops constituted convenient venues for interviewing women in traditional social roles who are not represented in the work force. In a study comparing the attitudes towards women in Kuwait and Qatar, Abdalla^12^ over-came the difficulty of house-to-house surveys by interviewing respondents in their work places.

To obtain a fairly representative national sample, we used the above methodologies to sample prospective respondents in these popularly accessed facilities of each of the governorates. This sampling methodology is feasible in Kuwait because of the small population, the urban residence, and the favorable education and employment circumstances. Hence, it is reasonable to assume that a fairly representative proportion of Kuwaiti nationals patronize the above popular urban places, and that they are literate enough in Arabic to complete uncomplicated questionnaires on their own.^11^

For the operational definition of QOL we accepted the WHO definition as the individuals' perception of life in the context of the culture and value system in which they live and in relation to their goals, expectations, standards and concerns.^6^ The measure focused on subjective QOL, as distinct from objective QOL.^13^

### The WHOQOL-BREF

The WHOQOL-BREF is a 26-item, self-administered, generic questionnaire that is a short version of the WHOQOL-100 scale.^6^ The response options range from 1 (very dissatisfied/very poor) to 5 (very satisfied/very good). Assessments are made over the preceding two weeks. It consists of domains and facets (or sub-domains). The items on “overall rating of QOL” (OQOL) and subjective satisfaction with health constitute the general facet on OQOL and health. The more popular model for interpreting the scores has four domains, namely, physical health (seven items), psychological health (six items), social relations (three items) and environment (eight items). Our analysis was based on this model. The domain scores of the WHOQOL-BREF can be computed in three ways. The first is a summation of the raw scores of the constituent items. The second and third ways consist of transforming the raw scores. In the second way, the raw scores are transformed into scores that range from 4-20, to be in line with the WHOQL-100 Instrument. The third way converts the 4-20 scores onto a 0-100% scale.^14^

### Data collection

The study took place in 2006-2007. The questionnaire was translated into Arabic by the method of back-translation. Ethical approval for the study was obtained from Kuwait University and the Kuwait Foundation for the Advancement of Science (KFAS). We obtained permission from the authorities of each study location to interview Kuwaiti nationals attending their facility. All participants gave verbal informed consent. The staff of a professional social research company was responsible for circulating and retrieving the questionnaires. These research assistants (RAs) were all Kuwaiti nationals, aged 21 to 27 years, who had previously undergone a 2-month course on research methods and interview techniques at Kuwait University. Pairs of RAs (one male, one female) worked in their own governorates of residence, so that they were familiar with the cultural environment. As a rule, prospective female respondents were approached by a female RA. To recruit prospective respondents, the RAs were positioned at the main entrance of a place, introduced themselves as Kuwaitis doing a research, and politely asked the nationality of the prospective participant. This was to ensure that only Kuwaiti nationals were recruited. Thereafter, the objectives of the study were explained and the subject was informed that no penalty would result from declining to participate. We used a systematic random sampling of one-in-three subjects (by gender). If a subject declined to participate, the next third person was approached. We continued in this way until we reached the population quota for the governorate. At the preliminary stage of the study, the RAs were trained in the use of the study's questionnaires in a one-week period. We did not compute inter-rater reliability because the questionnaires were all administered as self-report. Only subjects literate in Arabic were invited to participate. In all cases, the participants completed the questionnaire anonymously and privately. The RAs were nearby to offer assistance in clarifying the items. All subjects completed the Arabic translation of the questionnaire. Test-retest reliability was done by giving the questionnaire twice in a one-week period to 50 subjects who did not participate in the main study.

### Data analysis

Data were analyzed by SPSS version 11 (SPSS Inc, Chicago, Illinois). QOL domain scores (range, 4-20 and 0% to 100%) were generated by organizing the items into the four domains as recommended.^6^ Data for test-retest reliability were analyzed by intra-class correlation coefficient (ICC). The internal consistency for the full questionnaire and domains was assessed by Cronbach's alpha for the 3303 participants, with a recommended cut-off value of ≥0.7. The acceptability of the questionnaire was assessed by the missing values or proportion of respondents who failed to complete each item. A cut-off value of <2.5% is recommended for each item.^10^ The proportion of respondents scoring at the lowest level (floor effect) and the highest level (ceiling effect) for each item was assessed. This is a measure of sensitivity to change and how far the item can be assumed to be capturing the full range of potential responses in the population.^10^ Item internal consistency (IIC) and item discriminant validity (IDV), measured by the Pearson correlation, were assessed after controlling for item overlap in the corresponding scale. The IIC and IDV concern the relationship of each item to its hypothesized scale or domain. The IIC rule requires that the item should correlate r ≥0.4 with its adjusted scale score. For IDV, the item should have the highest correlation with its scale, in comparison with other scales in the questionnaire.^10^

The construct validity of the WHOQOL-BREF was assessed by exploratory factor analysis, using principal components analysis with orthogonal rotation for factors that have Eigen values above 1.^6^ Known-groups validity was assessed by testing the significant differences in domain scores between subjects who rated themselves as being well and those who rated themselves as being sick, using standardized effect size calculations. The relationship between the general facet and the domains was assessed by the Pearson correlation. Missing data were handled by excluding cases analysis by analysis. The level of statistical significance was set at 5%, and all tests were two-tailed.

## RESULTS

### Socio-demographic characteristics

Of the 3376 subjects who agreed to participate in the study, 73 questionnaires were voided because subjects did not complete over 20% of the items of the WHOQOL-BREF, as recommended by the WHOQOL Group.^6^ Hence we report data for 3303 subjects. The 3303 participants consisted of 44.8% (n=1479) men and 55.2% (n=1824) women, aged 16 to 87 years with a mean (SD) of 35.4 (11.9) years, with a proportionate representation of the six governorates. As in the Kuwaiti national general population, women were in the majority and 2.4% of the subjects were 65 years of age or older. The subjects were predominantly educated (59.9% had at least college education), employed in skilled work (58.4%), and married (60.8%).

### Descriptive statistics and internal consistency

The ICC for the test-retest statistic (0.95) was highly significant. Similarly, the internal consistency values for the full questionnaire and the domains met the 0.7 Cronbach's alpha value requirement (Table 1). With the exception of the item on sexual satisfaction (7.9%), the proportion of missing values ranged from 0.06% to <1% for 18 items, and from 1% to 1.8% for seven items. This shows that the contents of the questionnaire were generally acceptable to the respondents. Our rate of missing values for the item on sexual satisfaction is comparable to the 6.0% reported by the WHO, but higher than the 2.5% reported from Iran^4^ where the questionnaire was interviewer-administered. The mean (SD) scores for the items ranged from 3.39 (1.2) to 3.89 (0.99) for 23 items; it was 4.0 (1.1) for two items (mobility and transport), and 2.96 (0.98) for one item (negative feelings). With regard to the floor and ceiling effects, the total frequency of lowest scores was 4.7% (range, 2.2% to 8.9%), while the total frequency of highest scores was 24.9% (range, 6.8% to 42.2%). The comparable figures for the WHO report were 4.0% (range, 1.7% to 8.8%) and 17.5% (range, 10.1% to 35.2%), respectively.^6^

**Table 1:** Internal consistency of the WHOQOL-BREF for all participants, and test-retest reliability for non-participating subjects.

Questionnaire/domain QOL	No. of items	Cronbach's alpha	Split-half reliability
Internal consistency:			
WHOQOL-BREF (n=3303)	26	0.93	0.89
Physical health domain WHOQOL-BREF	7	0.80	
Psychological health domain	6	0.77	
Social relations domain	3	0.69	
Environment domain	8	0.83	

Test-retest reliability		(95% C.I.)*	
	26×2	
WHOQOL-BREF (n=50)		0.95 (0.94-0.97)	

* Intra-class correlation coefficient and 95% confidence interval.

### Item internal consistency and item discriminant validity

Of the 24 items that constitute the domains, 21 met the IIC requirement of correlation ≥0.4 (Table 2). Only the three negatively worded items (on pain, need for medical treatment and negative feelings) did not meet this requirement. However, these three items were among the 16 that met the IDV criterion of having a higher correlation with their corresponding domain more than other domains. Two more items had equal correlation with at least one more domain other than their corresponding domain. In the WHO 23-country report,^6^ it was noted that in 7 of 24 centers, the items on pain and the need for medical treatment were generally problematic in item-total correlations in the physical domain. In addition, poor item-total correlation (<0.3) was noted for negative feelings in one center.

**Table 2:** Item-internal consistency and item-discriminant validity: WHOQOL-Bref: Pearson's r>0.4*

WHOQOL items arranged by 4 domains	Physical health	Psychological health	Social relations	Environment
Physical health**				
Pain prevents activities	0.34	0.17	0.08	0.11
Enough energy for daily life	0.60	0.64	0.49	0.59
Able to get around	0.62	0.51	0.46	0.51
Satisfaction with sleep	0.57	0.54	0.49	0.56
Satisfaction with ADL	0.69	0.61	0.53	0.59
Satisfaction with work capacity	0.59	0.55	0.52	0.51
Need treatment to function	0.39	0.19	0.14	0.16

**Psychological health**				
How much enjoy life	0.48	0.63	0.47	0.59
Feel life meaningful	0.43	0.63	0.47	0.56
Able to concentrate	0.46	0.53	0.42	0.53
Accept bodily appearance	0.49	0.46	0.38	0.49
Satisfaction with self	0.59	0.58	0.63	0.59
How often negative feelings	0.26	0.27	0.22	0.26

**Social relations**				
Satisfaction with personal relationships	0.48	0.58	0.57	0.55
Satisfaction with sex life	0.45	0.49	0.45	0.44
Satisfaction with friends' support	0.39	0.44	0.49	0.52

**Environment**				
Feel safe in daily life	0.48	0.63	0.48	0.59
Healthy physical environment	0.40	0.59	0.45	0.59
Have enough money for needs	0.42	0.53	0.41	0.58
Satisfaction information for day-to-day life	0.46	0.53	0.41	0.56
Have leisure opportunity	0.39	0.44	0.35	0.46
Satisfaction living place	0.45	0.51	0.54	0.59
Satisfaction with access to health service	0.35	0.34	0.36	0.49
Satisfaction with transport	0.43	0.42	0.44	0.53

* Corrected for overlap of items in domain

** Items in bold belong to the corresponding domain

### Construct validity: factor analysis

In factor analysis with all the 26 items, four strong factors (i.e., each with >3 items) and one weak factor (with 3 items) emerged, accounting for 58.7% of the variance. Each item loaded highly (≥0.45) on the corresponding factor (Table 3). Interestingly, the four strong factors emerged exactly in the same sequence and with the same construct as in the WHO report. The three negatively worded items constituted the fifth factor. Of the 8 items in Factor I, five belong to the original physical health domain. It is noteworthy that the two general facet items (representing subjective well-being) were in this factor. Factor II is conceptually similar to the original psychological health domain because, of the five constituent items, three belong to psychological health. Factor III is conceptually similar to the original social relations domain because it contains the three items that define that domain. Factor IV appears to be a tighter definition of the environment domain because all the seven items were derived from that domain.

**Table 3:** Factor analysis using all 26 items of the WHOQOL-Bref

Factor label	Items of WHOQOL-Bref	Item loading	Eigen value	% Variance	Total variance
Factor I: General physical health and overall QOL	Ability to get around	0.71	10.2	39.1	58.7
	Energy for life	0.66			
	Satisfaction with general health	0.58			
	Satisfaction with work capacity	0.56			
	Accept bodily appearance	0.54			
	Activities of daily living	0.54			
	Overall rating of QOL	0.49			
	Satisfaction with sleep	0.46			

Factor II: Psychological Health	Feel life meaningful	0.67	1.7	6.6	
	Enjoyment of life	0.67			
	Physical environment health	0.65			
	Feel safe in daily life	0.65			
	Able to concentrate	0.52			

Factor III: Social relations	Satisfaction with personal relations	0.68	1.2	4.7	
	Satisfaction with sex	0.64			
	Satisfaction with self	0.63			
	Satisfaction with support from friends	0.58			

Factor IV: Environment	Satisfaction with access to health service	0.73	1.2	4.5	
	Satisfaction with transport	0.57			
	Enough money for needs	0.56			
	Satisfaction with condition of place of living	0.53			
	Opportunity for leisure activities	0.48			
	Satisfaction with available information	0.47			

Factor V: Health care needs	Pain	0.86	1.0	3.9
	Need for medical treatment	0.79		
	Negative feelings	0.52		

Kaiser-Meyer-Olkin measure of sampling adequacy=0.95

### Known-groups validity and the relationship of domains with the general facet

Using the 0-100 scale scores in effect size calculations (effect size, 95% confidence interval), we found that those who rated themselves as being well had significantly higher scores in all domains: physical health (0.50, 0.43-0.57), psychological health (0.36, 0.29-0.44), social relations (0.31, 0.24-0.38) and environment (0.30, 0.23-0.38) domains (*P*<.0001). Finally, the general facet score was highly significantly correlated with the scores for all the domains: physical health (r=0.63), psychological health (r=0.66), social relations (r=0.53) and environment (r=0.61) domains (*P*<.0001).

## DISCUSSION

A major limitation of the study is that the subjects were not a representative house-to-house sample of the general population. In particular, those who did not attend the study locations were not represented. This limitation is inherent in the socio-cultural circumstances of the country.^11^,^12^ In view of this limitation, it was not practically possible to keep track of the number of refusals and their characteristics. However, it is reasonable to assume that our nationwide sample had adequate characteristics to estimate the psychometric properties of the WHOQOL-BREF.^10^

In line with the hypothesis that the reliability and validity indices are similar to those in other populations, the Arabic translation of the WHOQOL-BREF generally met the statistical criteria for the reliability issues investigated. In particular, our findings were similar to those of the WHO 23-country report.^6^ There are three notable discrepancies: the relatively high proportion of missing values for the item on sexual satisfaction, the failure of the three negatively worded items to meet the IIC requirement, and the fact that only 16 items met the IDV requirement. A relatively minor discrepancy is the fact that the alpha coefficient for the social relations domain (0.69) was just short of the 0.7 mark. These discrepancies are well known in the literature. The sexual item is usually a problem, where respondents complete the questionnaire on their own, as in our study. In a Taiwanese study, the rate of missing values ranged from 0.9% to 7.7%.^15^ In general population studies from Poland and Iran, only the social relations domain failed to meet the required value for internal consistency.^4^,^16^ This problem is related to the fact that the domain has only three items.

We could not attribute the poor IIC performance of the three negatively worded items to translation problems because they met the IDV requirement and the problem was also evident in some centers in the WHO report. Furthermore, the items loaded very highly (>0.5) in the factor analysis. The issue of poor performance in IIC and IDV has been noted in other ways by studies that used the Rasch and item response theory models,^17^,^18^ and is attributable to the multidimensional nature of the instrument.^18^

Our most impressive findings were for construct and known-groups validity. Our first four factors are a fair representation of the original WHO data^6^ and have been replicated by others.^19^,^20^ In a Sudanese study, the following factors were replicated: social relations, environment, and the small factor containing the three negatively worded items. Furthermore, in confirmatory factor analysis, the four-domain model had the best fit indices.^9^ Our results thus support the impression that the same perceptions of the WHOQOL-BREF are found across cultures and disease conditions,^19^ a cardinal requirement for a cross-culturally useful questionnaire. Furthermore, the two general facet items loaded highly on Factor I. This indicates the importance of the general facet as a first deconstruct of the QOL construct,^21^ and hence it is a fair representation of the idea of global QOL/ subjective well-being.^22^,^23^ In support of this point, the general facet was highly significantly correlated with the four domains.^6^

In conclusion, the Arabic translation of the WHOQOL-BREF has high reliability and validity indices. It represents the same constructs across cultures.^19^ Our validity data support the impression that the WHOQOL-BREF is a cross-culturally valid generic instrument that is composed of parts that address the main issues of the subjective QOL construct in medicine, namely HRQOL, contextual issues and subjective well-being.^8^ A possible area needing further development is the phrasing of negatively worded items, perhaps by having them rated in a positive direction as the other items.
